# Mobile critical care recovery program (m-CCRP) for acute respiratory failure survivors: study protocol for a randomized controlled trial

**DOI:** 10.1186/s13063-018-2449-2

**Published:** 2018-02-07

**Authors:** Sikandar Khan, Ashok Biju, Sophia Wang, Sujuan Gao, Omar Irfan, Amanda Harrawood, Stephanie Martinez, Emily Brewer, Anthony Perkins, Frederick W. Unverzagt, Sue Lasiter, Ben Zarzaur, Omar Rahman, Malaz Boustani, Babar Khan

**Affiliations:** 1 Division of Pulmonary, Critical Care, Sleep and Occupational Medicine, Department of Medicine, Indiana University School of Medicine, Indianapolis, IN USA; 2 IU Center of Aging Research, Regenstrief Institute, Indianapolis, IN USA; 3 Department of Psychiatry, Indiana University School of Medicine, Indianapolis, IN USA; 4 Center of Health Innovation and Implementation Science, Center for Translational Science and Innovation, Indianapolis, IN USA; 5 Sandra Eskenazi Center for Brain Care Innovation, Eskenazi Hospital, Indianapolis, IN USA; 6 Department of Biostatistics, Indiana University School of Medicine, Indianapolis, IN USA; 7 Aga Khan University, Karachi, Pakistan; 8 School of Nursing and Health Studies, University of Missouri, Kansas City, MO USA; 9 Department of Surgery, Indiana University School of Medicine, Indianapolis, IN USA; 10 Division of Geriatrics and General Internal Medicine, Department of Internal Medicine, Indiana University School of Medicine, Indianapolis, IN USA

**Keywords:** Delirium, Physical activity, Cognitive training, Cognitive impairment, Critical care, ICU survivorship, Quality of life, Post-intensive care syndrome

## Abstract

**Background:**

Patients admitted to intensive care units (ICU) with acute respiratory failure (ARF) face chronic complications that can impede return to normal daily function. A mobile, collaborative critical care model may enhance the recovery of ARF survivors.

**Methods:**

The Mobile Critical Care Recovery Program (m-CCRP) study is a two arm, randomized clinical trial. We will randomize 620 patients admitted to the ICU with acute respiratory failure requiring mechanical ventilation in a 1:1 ratio to one of two arms (310 patients per arm) – m-CCRP intervention versus attention control. Those in the intervention group will meet with a care coordinator after hospital discharge in predetermined intervals to aid in the recovery process. Baseline assessments and personalized goal setting will be used to develop an individualized care plan for each patient after discussion with an interdisciplinary team. The attention control arm will receive printed material and telephone reminders emphasizing mobility and management of chronic conditions. Duration of the intervention and follow-up is 12 months post-randomization.

Our primary aim is to assess the efficacy of m-CCRP in improving the quality of life of ARF survivors at 12 months. Secondary aims of the study are to evaluate the efficacy of m-CCRP in improving function (cognitive, physical, and psychological) of ARF survivors and to determine the efficacy of m-CCRP in reducing acute healthcare utilization.

**Discussion:**

The proposed randomized controlled trial will evaluate the efficacy of a collaborative critical care recovery program in accomplishing the Institute of Healthcare Improvement’s triple aims of better health, better care, at lower cost. We have developed a collaborative critical care model to promote ARF survivors’ recovery from the physical, psychological, and cognitive impacts of critical illness. In contrast to a single disease focus and clinic-based access, m-CCRP represents a comprehensive, accessible, mobile, ahead of the curve intervention, focused on the multiple aspects of the unique recovery needs of ARF survivors.

**Trial registration:**

NCT03053245, clinicaltrials.gov, registered February 1, 2017.

**Electronic supplementary material:**

The online version of this article (10.1186/s13063-018-2449-2) contains supplementary material, which is available to authorized users.

## Background

With advances in medical technology and an aging population, the United States now has millions of acute respiratory failure (ARF) survivors [1, 2]. Due to illness severity, physiologic derangements, procedural and pharmacologic interventions, invasive mechanical ventilation, and acute brain dysfunction, ARF survivors are predisposed to experience post-intensive care syndrome (PICS). PICS is defined as a cluster of debilitating symptoms characterized by physical and cognitive impairment as well as symptoms of depression and anxiety, with a negative impact on their overall quality of life (QOL) [3–15]. PICS is prevalent, with greater than 50% of ARF survivors affected, and impairs recovery after hospital discharge and can persist for years [16, 17].

After an ICU stay, patients are referred to their primary care provider for follow-up care. Primary care providers often do not have appropriate resources to identify or treat PICS symptoms, resulting in diminished QOL and long-term disability for patients [18, 19–22]. As mentioned above, more than 50% of ARF survivors suffer from functional or cognitive disability, major or minor depression, and anxiety.

## Rationale for a mobile critical care recovery program (m-CCRP)

Although community and rehabilitation services are available to ARF survivors, the fragmented nature of our healthcare system makes it challenging to coordinate and direct care to meet the full needs of the patient. This state of disorganized care for post-ICU PICS patients hinders their chance for optimal recovery and return to pre-hospitalization baseline (including employment) [18, 19–25]. Access to a m-CCRP empowered with tools and support to integrate and connect essential resources has the potential to enhance the recovery of ARF survivors.

Existing literature on recovery services for ARF survivors does not outline a clear path to manage the multiple comorbidities of PICS. Many of the studies on the subject have been performed outside the US, limited by small sample sizes, and failed to address the multidimensional nature of PICS or had an inadequate follow-up periods [25–27].

We designed our study based on results of the IMPACT and PREVENT randomized controlled trials, where interdisciplinary care management for participants suffering from depression and dementia, respectively, was associated with improved outcomes [28, 29]. Participants had improved QOL (IMPACT) and reduced behavioral and psychological symptoms (PREVENT). In the GRACE trial, a care team study using protocols for home-based management, participants had lower emergency room and hospital visits, and improved QOL scores in general health, vitality, social function, and mental health [30]. Given the potential for psychosocial and behavioral effects of our trial, we used an attention-control comparator arm to test the specific effects of the intervention [31].

## Conceptual model

**Fig. 1:**
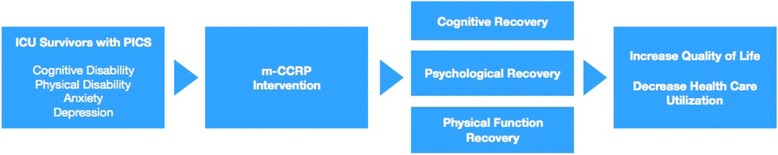
Conceptual model for m-CCRP study. *ARF* acute respiratory failure, *PICS* post-intensive care syndrome

**Fig. 2:**
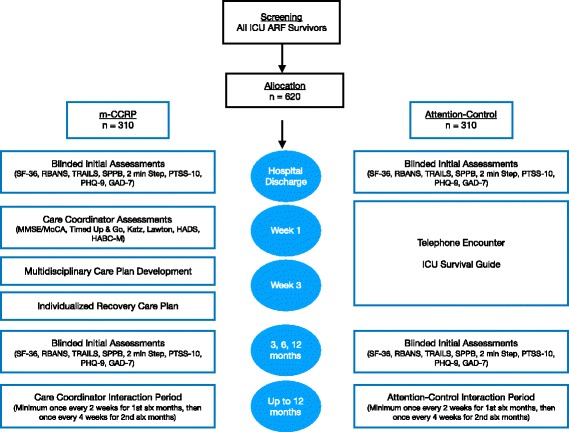
m-CCRP study timeline. *ARF* acute respiratory failure, *GAD-7* Generalized Anxiety Disorder Scale-7, *HABC-M* Healthy Aging Brain Center monitor, *HADS* Hospital Anxiety and Depression Scale, *Katz* Katz Index of Independence in Activities of Daily Living, Lawton Lawton Instrumental Activities of Daily Living, *m-CCRP* mobile critical care recovery program, *MMSE* Mini-mental Status Exam, *PHQ-9* Patient Health Questionnaire-9, *RBANS* Repeatable Battery for Assessment of Neuropsychological Status, *SF-36* 36-Item Short-Form Survey (Quality of Life), *SPPB* Short Physical Performance Battery

Our study is aimed at developing a mobile multidisciplinary intervention (m-CCRP) led by a nurse care coordinator to meet the biopsychosocial needs of ARF survivors with physical, psychological, and cognitive impairment (Fig. 1). The care coordinator will implement the intervention at the physical residence of the patient and seek to improve recovery through input from an interdisciplinary team. The interdisciplinary team is composed of a critical care physician, a health services scientist, an ICU nurse, a psychologist, and other consultants as needed to guide the recovery process over the course of 12 months (Fig. 2). The recovery process will be informed through dynamic feedback through process measurement tools and care coordination support software.

## Objectives

We sought to answer the following question: Does a m-CCRP, offering multidisciplinary individualized care plans, improve the QOL of acute respiratory failure survivors at 12 months?

### Primary aim

To assess the efficacy of m-CCRP in improving the QOL of ARF survivors compared to attention control at 12 months post hospital discharge.

### Secondary aims

Our study has the following secondary aims:

1. To evaluate the efficacy of m-CCRP in improving cognitive, physical, and psychological function of ARF survivors at 12 months post hospital discharge when compared to attention control.
2. To evaluate the efficacy of m-CCRP in reducing healthcare utilization, defined as time from enrollment to emergency department visits and/or hospital re-admission, by ARF survivors as compared to attention control at 12 months post hospital discharge.

## Methods/design

### Type of trial

The m-CCRP study is a two-arm randomized parallel-group superiority clinical trial. Patients admitted to the ICU and diagnosed with ARF are eligible. A total of 620 patients will be randomized (1:1) to one of two arms (310 patients per arm): m-CCRP intervention vs. attention control.

We chose an attention-control (rather than usual care) arm to better assess the efficacy of our intervention beyond behavior modification that is likely to occur within the controlled environment of the trial.

**Fig. 3:**
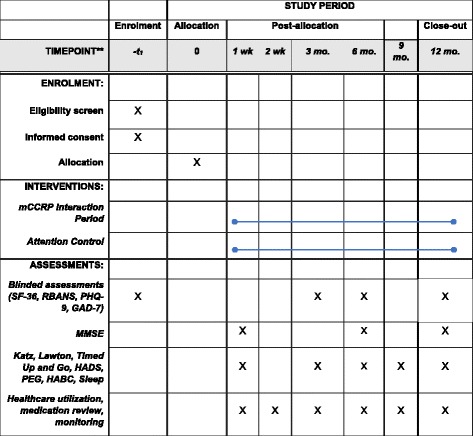
Standard Protocol Items (SPIRIT) for the m-CCRP Trial. *GAD-7* Generalized Anxiety Disorder Scale-7, *HADS* Hospital Anxiety and Depression Scale, *Katz* Katz Index of Independence in Activities of Daily Living, *Lawton* Lawton Instrumental Activities of Daily Living, *m-CCRP* mobile critical care recovery program, *MMSE* Mini-mental Status Exam, *PEG* Pain Scale, *PHQ-9* Patient Health Questionnaire-9, *RBANS* Repeatable Battery for Assessment of Neuropsychological Status, *SF-36* 36-Item Short-Form Survey (Quality of Life)

The trial protocol was written according to the Standard Protocol Items: Recommendations for Interventional Trials Statement (SPIRIT). A SPIRIT table is provided (Fig. 3), and a SPIRIT checklist is included in Additional file 1.

### Study setting

Patients will be recruited from local tertiary care hospitals (Indiana University Health Methodist and University Hospitals and Sidney & Lois Eskenazi Hospital). Eskenazi Hospital is a busy 618-bed, urban, safety net hospital with 44 ICU beds (22 surgical, 22 medical), averaging 120 ICU admissions per month. The demographics of this ICU are a mean age of patients 53.7 years, 45% African American, 47% female, 29% aged 60 years or older, and 16% with Medicaid insurance. Methodist Hospital is an urban, quaternary care hospital with 802 beds. Patients will be recruited from three mixed medical and surgical ICU units (65 beds). These ICU units have 153 monthly admissions of which 48% are females, 47% elderly (age ≥ 60 years), 31% African Americans, and < 1% with Medicaid insurance. University hospital is a 257-bed, urban, tertiary care hospital with 18-bed medical and 18-bed surgical ICUs admitting 50 ICU patients every month. Given the close affiliation of all centers with the Indiana University School of Medicine, and the approval of the study under a single Institutional Review Board (IRB), the need for a coordinating center or steering committee is not anticipated.

### Recruitment of participants

The target population for our trial are adults aged 18 years and older who have been admitted to the medical or surgical ICUs and are on invasive mechanical ventilation.

### Inclusion criteria

Inclusion criteria for our study are (1) age 18 years or older; (2) hospitalized in the ICU; (3) diagnosed with ARF requiring greater than or equal to 24 hours of invasive mechanical ventilation; (4) discharged to home, skilled nursing facility, sub-acute rehabilitation care, or long-term acute care; (5) English speaking; (6) able to consent either in person or through legally authorized representative; and (7) access to a telephone.

### Exclusion criteria

Patients are excluded from the study if they meet any of the following criteria: (1) hospitalized in a non-ICU ward; (2) diagnosis of cancer with life expectancy less than 1 year; (3) admitted with ischemic or hemorrhagic stroke, traumatic brain injury (based on the treating physicians’ notes or evidence of brain injury on neuroimaging), or undergoing neurosurgery; (4) history of dementing illnesses and other neurodegenerative diseases such as Alzheimer disease, Parkinson disease, or vascular dementia; (5) unable to complete study questionnaire due to severe hearing loss; (6) legally blind; (7) pregnant (assessed by urine or serum pregnancy test) or nursing; (8) living outside the greater Indianapolis area; (9) recent history of alcohol or substance abuse; (10) status post tracheostomy and not eligible for a speaking valve; and (11) incarcerated at the time of study enrollment.

### Screening, enrollment, and subject randomization

Research staff check the electronic medical records of ICU patients daily for eligibility. After eligibility is confirmed, the patient and/or their legal representative are approached for informed consent. In cases where the participant is not able to provide consent at the time of screening, consent is obtained from the surrogate, and the participant is approached for consent once they are able to consent, and prior to discharge. The study participant is then randomized via a computer-generated randomization scheme to either the intervention or attention control arm (permuted block sizes of 6 and 8), stratified by hospital.

### Pre-intervention phase

Forty-eight hours prior to hospital discharge and after two consecutive Confusion Assessment Method negative evaluations, research assistants perform blinded in-person initial evaluations to assess QOL, cognition, physical function, depression, and anxiety [32–40]. Details regarding the assessments and outcome measures are discussed under ‘Outcome assessments’ below.

### Intervention phase

#### m-CCRP intervention

Upon randomization, and after hospital discharge, the care coordinator will contact patients randomized to the intervention arm. The care coordinator is a registered nurse whose scope of practice includes health coaching, case managing, and liaising with community resources and nursing care providers. The care coordinator reviews hospital discharge and rehabilitation plans, identifies the primary care provider, obtains the approval of the primary care provider to co-manage post ICU care of the eligible patients, and schedules a face-to-face visit with the patients at their place of discharge (home, sub-acute rehabilitation facility/skilled nursing facility or long-term acute care).

##### First visit

The first visit will take place within 1 week of hospital discharge. The coordinator will assess the patient’s cognitive, physical, and psychological status. The coordinator will perform a needs assessment for both the patient and the caregiver, reconcile all prescribed and over-the-counter medications, make note of all scheduled and recommended appointments made at discharge with specialists and therapists, and assess for community-based needs. Personalized recovery goals will be discussed with patients and their caregivers to inform the care plan.

Initial and follow-up visits will be documented in the care coordination support software, EMR-ABC [41].

##### Individualized care plans

Using the assessments and information obtained at the first visit, the coordinator will collaborate with the interdisciplinary team and primary care physician to finalize the individualized care plan (Fig. 2). Patients that may benefit from specialty care will be recommended for specialty evaluation and co-management. If necessary, the patient will be referred for a more extensive cognitive and psychological evaluation at the local mental health practices. This decision will be jointly reached by the care coordinator, the m-CCRP team members, the patient, their caregiver, and the patient’s primary care provider. Finally, a second face-to-face visit within 2 weeks of the first visit will be arranged.

##### Second visit

During the second visit, the coordinator will review the individualized recovery care plan with both the patient and the informal caregiver. This process will include (1) understanding the diagnoses; (2) process of monitoring the patient’s recovery; (3) implementation of the appropriate care recovery protocols; (4) distribution and explanation of the corresponding educational recovery handouts (patient and informal caregiver); and (5) connection to in-home services and community resources.

##### Interaction period

The 12-month interaction period will consist of regular contact between the care coordinator, patient, and informal caregiver via face-to-face home or clinic visit, phone contact, email, fax, or mail. During the first 6 months, the minimum amount of contact will be once every 2 weeks. For the last 6 months, the minimum amount of contact will be once a month. During these interactions, the coordinator will answer questions generated from previous visits, collect patient and caregiver’s feedback, review and reconcile medications and discuss adherence, review specialists and therapists appointments and adherence to care plans, have the patient and the caregiver complete the HABC Monitor [42] to trigger or modify the use of specific care recovery protocols, and facilitate the caregiver’s access to appropriate community resources. The care coordinator will work with the interdisciplinary team, the patient’s primary care provider, and specialists to incorporate patient/caregiver feedback for any revisions needed to the individualized care plan (Fig. 2).

##### Care protocols

The care coordinator will implement care protocols developed by our interdisciplinary team. These protocols have been utilized in the Critical Care Recovery Center at our institution. These care plans address cognition, physical function, personal care, mobility, sleep disturbance, depression, anxiety, agitation or aggression, delusions or hallucinations, caregiver stress/physical health, driving safety, nutrition, and medication adherence.

##### Acute care transition

If a patient develops an acute illness requiring hospitalization, the team activates the acute care transition phase. The coordinator contacts the hospital team with relevant information about the patient’s cognitive, physical and psychological symptoms, and the patient’s medication list. Upon hospital discharge, the coordinator conducts a home visit within 72 hours.

##### Post intervention

At the end of 12 months, every patient will be transitioned to receive full care by their primary care provider.

### Needs assessments during intervention

During the first visit, 1 week after hospital discharge, the coordinator will assess the patient’s cognitive, physical, and psychological status using the Mini-mental State Examination (MMSE) [43] and/or Montreal Cognitive Assessment (MoCA), Timed up and Go test [44], activities of daily living (Katz scale) [45], instrumental activities of daily living (Lawton Scale) [46], Hospital Anxiety and Depression Scale (HADS) [47], and the Healthy Aging Brain Care Monitors (HABC-M) [41]. Pain will be assessed by the PEG scale [48], and sleep symptoms [49] measured are also assessed. The HABC-M will also be used to assess caregiver stress. Initial and follow-up visits will be documented in care coordination software, the EMR-ABC.

### Attention control

After randomization, subjects assigned to the attention control group will be contacted by the study research coordinator to ensure the patient has a primary care physician appointment at hospital discharge. Patients will receive a guide for ICU survivors containing phone numbers for relevant community resources, education on caregiver coping skills, and legal and financial advice. An independent care coordinator/research assistant, not involved in any outcome assessments, will contact those in the attention control group by phone. The calls will be scripted so as to inquire about the patient’s cognitive, physical, emotional, and general well-being. The frequency of the calls will mirror the study flow of the intervention group visits (within 1 week post discharge, within 2 weeks post first phone call, afterwards twice per month for first 6 months, and once per month for the next 6 months). Patients will be directed to use the information provided in the ICU survivor guide to connect with resources. For those without access to the guide, the relevant phone numbers will be provided by telephone and a new guide mailed within 24 hours of the phone call.

Should an ICU survivor voice concern for any life-threatening symptoms (severe chest pain, shortness of breath, bleeding, suicidality), the research assistant will advise them to present to the emergency room or call emergency medical services, and notify the study investigator. The primary investigator (a critical care physician) remains on call for the study, as does a geriatric psychiatrist.

### Outcome measures during the study

#### QOL outcomes

Patient’s health-related QOL will be assessed using the Medical Outcome Study Short Form (SF-36). This scale has eight components (physical functioning, role-physical, bodily pain, general health, vitality, social functioning, role-emotional, and mental health) that are aggregated into a Physical Component Summary (PCS) and a Mental Component Summary (MCS) [32, 33]. Changes that differ between groups by 2 or more points on a scale of 0 to 100 have been shown to be clinically meaningful.

#### Cognitive assessment

We will use the Repeatable Battery for the Assessment of Neuropsychological Status (RBANS) [35], and the Trail Making Test Part A and B. The RBANS was developed to identify and characterize cognitive functioning for adults aged 12–90 years. It takes 30 minutes to administer and yields scaled scores for five cognitive domains (Immediate Memory, Visuospatial Construction, Language, Attention, and Delayed Memory). The RBANS has excellent reliability and has been validated across various clinical samples. The RBANS has four parallel versions, which will be counterbalanced across occasions of measurement.

#### Physical performance

Physical recovery will be assessed via the Short Physical Performance Battery (SPPB), a validated objective assessment [36]. The SPPB yields a performance score of 0–12 (0–4 poor, 5–7 intermediate, 8–12 good). A difference of 1 point indicates significant change in function. Physical training and cardiovascular fitness will be assessed using the 2-minute step test, a validated measure of aerobic capacity.

#### Depression and anxiety symptoms

We will use the Patient Health Questionnaire-9 (PHQ-9) [37, 38] and Generalized Anxiety Disorder Scale (GAD-7) [39, 40] to determine the impact of the intervention on ARF survivors’ mood and anxiety. The PHQ-9 is a nine-item self-report depression scale with a total score from 0 to 27 and the GAD-7 is a seven-item self-report anxiety scale with a total score from 0 to 21. Both of these scales are derived from the Patient Health Questionnaire, have good internal consistency and test–retest reliability, as well as convergent, construct, criterion, procedural, and factorial validity for the diagnosis of major depression and general anxiety disorder. We will use the PTSS-10 questionnaire to assess for post-traumatic stress syndrome symptoms. The delirium experience questionnaire will be used to elicit delirium recall.

#### Acute healthcare utilization

In addition to patient reported emergency department and hospital admission data, we will use the local data-warehouse to capture all of the data needed to determine utilization. Furthermore, we will also use the data from the Indiana Network for Patient Care (INPC) to complement any data use outside of our health system. INPC is the primary health information exchange in the state of Indiana and it provides data for acute care services from several major healthcare systems within the state of Indiana. We will determine the number of emergency department visits and the number of re-hospitalizations within 12 months of the index discharge (study follow-up period) as well as the diagnoses associated with each utilization episode.

#### Permitted treatments and adherence

Participation in the trial will not preclude any other care deemed appropriate by the patient’s treating physicians. Visits and contact time with the participants will be recorded by the care coordinator as a means of monitoring adherence to the intervention protocols. Participants randomized to one arm of the trial will not cross over to another. Participants who wish to decrease the frequency of their follow-up due to improved health will still receive the minimum study contact as per protocol.

#### Blinding

Research assistants will perform blinded in-person outcomes assessments similar to the baseline assessment using the same instruments (SF-36, RBANS, SPPB, PHQ-9, GAD-7) at 3, 6, and 12 months. We will employ multiple techniques to ensure concealment of outcomes assessment [50, 51]. Our research assistants will be trained not to inquire about study assignments. They will be conducting structured assessments that do not provide room for qualitative interviewing that should prevent unblinding. They will not be involved in study assignments and treatment administrations. Subjects will be instructed not to discuss their therapy with the research assistants. Should unblinding occur, other research assistants without knowledge of the participant’s allocation will be utilized for assessments.

#### Data collection

Prior to trial initiation, study personnel will undergo training sessions on data collection and are individually tested on data entry as well as outcomes assessments.

Study data is collected and managed using Research Electronic Data Capture (REDCap) tools hosted at Indiana University [52]. REDCap is a secure, web-based application designed to support data capture for research studies, providing (1) an intuitive interface for validated data entry; (2) audit trails for tracking data manipulation and export procedures; (3) automated export procedures for seamless data downloads to common statistical packages; and (4) procedures for importing data from external sources.

#### Data dissemination

Access to the final dataset will be retained with the study investigators. Compensation for the trial, including harm, is not intended. Results of the trial are intended for publication in a peer-reviewed journal by the authors, and assistance of professional writers is not expected. Results will be shared with participants of the study by e-mail, and within our hospital system. Granting full access to the protocol or participant-level dataset is not intended.

#### Monitoring

Monitoring will be performed by an independent data safety monitoring board (DSMB) consisting of three members with expertise in critical care, clinical geriatrics, clinical trial methodology, and biostatistics. The DSMB will receive trial data from the biostatistician and choose to continue the study as planned, change the study protocol, or stop the trial early for harm. The DSMB will review trial data with the m-CCRP statistician in a closed session upon enrollment of 30 patients, and at least once every 12 months. DSMB members will delete or shred any files they receive for the review meetings. Meeting minutes and related data from each DSMB meeting will be retained in a secured file for inspection by regulatory authorities.

#### Adverse events

Clinical outcomes, including death, will be systematically tracked and included in the safety and efficacy analyses for the study. Death will not be recorded as an adverse event unless the investigator believes the event may have been related to the study protocol. Other clinical outcomes tracked include hospital or ICU readmissions, depressive symptoms, anxiety, agitation and behavioral disturbances, and fall and mobility problems, and will be categorized as adverse events if related to the study protocol. Serious adverse events include death, life-threatening episode requiring immediate intervention, inpatient hospitalization, persistent or significant incapacitation, and an episode requiring intervention to prevent permanent impairment/damage.

The study manager will monitor adverse events, and the principal investigator will be notified within 24 hours of identifying an occurrence. Serious and unexpected adverse events will be reported to the IRB, safety officer, DSMB, and NIH. Non-serious adverse events will be reported to the IRB as part of a continuing review. Unanticipated problems will also be reported.

#### Stopping guidelines

The trial can be prematurely paused or closed by the DSMB in order to evaluate safety information from the study, or if there is evidence of harm in the study.

#### Statistical analysis

We will compare randomization results to pre-planned randomization schedule to ensure randomization integrity. To verify the comparability of the randomized groups, patients’ baseline characteristics between the intervention and the usual care group will be compared using *t-tests* or Wilcoxon rank-sum tests for continuous variables and χ^2^ tests for categorical variables. All analyses will be conducted using SAS 9.4 software.

##### Primary aim

Mixed effects models will be used with repeated SF-36 PCS and MCS collected at baseline, and at 3, 6, and 12 months as the outcome measures, using group, time, and interactions between group and time as independent variables while adjusting for randomization stratum and other potential baseline covariates. An unstructured variance–covariance matrix will be used in the mixed effects models to adjust for the potential correlations among PCS or MCS obtained from the same individual over time. Post-hoc comparisons using linear contrasts will be conducted following a significant interaction between group and time to determine the earliest time when a group difference can be detected. Parameter estimation and inference for the mixed-effects models are conducted using the maximum likelihood approach, which yields robust parameter estimation and inference under many missing data mechanisms [53]. We will also include additional covariates to examine whether patients’ characteristics, severity of illness, or prior medical comorbidities are associated with the changes in QOL measures.

##### Secondary aim 1

Mixed effects models will be used with repeated cognitive (RBANS), physical (SPPB), depression (PHQ-9), or anxiety (GAD-7) measures collected at baseline, and at 3, 6, and 12 months as the outcome measures, using group, time, and interactions between group and time as independent variables while adjusting for randomization stratum and other potential baseline covariates, similarly to the analysis plan described for the primary aim. In addition, we will use mixed effect models with PCS and MCS as the dependent variables and cognitive, physical, and psychological functions as the independent variables to determine how changes in specific functional domains relate to changes in health-related QOL.

##### Secondary aim 2

Time from enrollment to emergency department visits and hospital readmissions will be used as the outcome variables in Cox’s proportional hazards models and group as the independent variable while adjusting for randomization stratum and other covariates. Patients who are followed to 12 months without experiencing any event will have their event time censored at 12 months; patients who died or were lost to follow-up will have their observation time censored at the time of death or date of last contact. Recurring events will be modeled using the Andersen–Gill model for multiple events using elapsed times with robust variance [54]. We will examine the proportional hazard assumption by including the interaction between the group indicator variable and time. We will also include other patients’ characteristics, illness severity and other comorbidities in the model to determine whether intervention effect is affected by other covariates.

##### Sample size justification

Based on our preliminary data in this patient population, we anticipate that 16% will die during follow-up and another 20% will be lost to follow-up. Thus, we estimate that we will have 400 patients completing the 12-month follow-up (200 patients per group). A previous study in ICU survivors reported a mean PCS increase from 29.0 (SD = 13.4) to 35.4 (SD = 11.8) and mean MCS increase from 44.6 (SD = 10.8) to 45.8 (SD = 10.6) [55]. Assuming patients in the usual care group follows the trajectory reported in Sukantarat et al. [55], 200 patients per group with completed follow-up data will provide 80% power to detect a difference of 0.28 SD in mean PCS or MCS at the 12-month follow-up using a two-sample *t-*test at α = 0.05. The projected effect size translates to approximately 3.3 points higher in PCS and 3.0 points higher in MCS for patients in the intervention group compared to those in the attention control at the end of intervention using the standard deviations reported in Sukantarat et al. [55]. Based on previous literature, we assume a baseline mean RBANS score of 79 (SD 11.85) [56]. The sample size of 200 patients per group will provide 80% power to detect a difference of 3.3 in RBANS scores between the two groups at 12 months assessment using a two-sample *t*-test at α = 0.05. Similarly, with a baseline mean SPPB score of 6.0 (SD = 2.5) in this patient population [36], our sample size will yield 80% power to detect a difference of 0.7 in SPPB scores during the 12-month intervention period. We are also powered to detect 0.28 SD in reduction in PHQ-9 and GAD-7 scores at the end of intervention. For power estimation of acute care utilization, we will have access to the electronic medical records of all enrolled patients (n = 620). Assuming a probability of 40% for emergency room visit or hospitalization for patients in the usual care group during the 12-month follow-up, we will have 80% power to detect a hazard ratio of 0.64 or lower for reduction in emergency room visits or hospitalization using log rank test at α = 0.05.

## Discussion

The proposed randomized controlled trial will evaluate the efficacy of a collaborative critical care recovery program in accomplishing the Institute of Healthcare Improvement’s triple aims of better health, better care, at lower cost [57]. Our study is innovative in several ways. We have developed a collaborative critical care model to promote ARF survivors’ recovery from the physical, psychological and cognitive impacts of critical illness. In contrast to a single disease focus and clinic-based access, m-CCRP represents a comprehensive, accessible, mobile, ahead of the curve intervention focused on the multiple aspects of the unique recovery needs of ARF survivors.

Previous studies in other conditions found that disconnecting the needs assessment from its subsequent management reduced the impact of such assessment [38]. The m-CCRP study couples comprehensive evaluation of ARF survivors with an interdisciplinary management program and delivers it in a bundled plan of care. At the same time, the individual goals of the patients and caregivers will be incorporated to form an individualized care plan. The care coordinator will identify and work towards ARF survivors’ and caregivers’ target goals by delivering individualized care plans and by tracking effectiveness of intervention. This will enhance survivors’ retention in the program and will provide regular feedback. Our care coordination support software [42] and the Healthy Aging Brain Care monitors will provide dynamic and actionable feedback to assess ARF survivors’ recovery in a timely manner. The resultant adjustments in the care plans are highly desirable for the frequently changing acute needs of ARF survivors, compared to the relatively stable chronic care needs of older adults.

Finally, m-CCRP will deliver care to the patient at home, rehab facility, or at their primary care or specialists offices. This obviates the need for excess travel by recovering ARF survivors.

As with all studies, we anticipate that there will be potential problems. First and foremost, recruitment in clinical trials is challenging in the ICU. Our team has developed an ICU research infrastructure that has allowed us to successfully recruit ICU patients as previously mentioned in our preliminary work.

Secondly, a longer follow-up poses a challenge in retention of subjects. We plan to mitigate withdrawals from both groups through several mechanisms. Quality control procedures allow us to monitor subjects’ perceptions about the risks and benefits of participation. We will recruit research personnel representatives of the target population by sex and race and provide training to work with vulnerable research populations. Retention will be periodically measured. If retention drops below 80%, we will have study staff follow-up with the study participants to troubleshoot issues and provide coaching. Gift card incentives will be instituted using fair subject payments to maximize participation.

While the proposed intervention has multiple components, the study is not designed to test which of these components is most efficacious. Caring for PICS patients is a multidisciplinary and multidimensional responsibility, and we do not believe that isolating one component is likely to improve care of these patients. Our research team’s capacity to design, deliver and measure outcomes for a multi-component intervention is one of our key strengths.

## Trial status

Enrollment is ongoing. Recruitment started in March 2017 and is expected to conclude January 2020. Target enrollment for the study is 620 subjects.

## Protocol version

Version 1.0, 09182016.

## Additional file

Additional file 1: SPIRIT 2013 Checklist: Recommended items to address in a clinical trial protocol and related documents^a^. ^a^It is strongly recommended that this checklist be read in conjunction with the SPIRIT 2013 Explanation & Elaboration for important clarification on the items. Amendments to the protocol should be tracked and dated. The SPIRIT checklist is copyrighted by the SPIRIT Group under the Creative Commons “Attribution-NonCommercial-NoDerivs 3.0 Unported” license. (DOCX 24 kb)

## Abbreviations

ARF: acute respiratory failure
GAD-7: Generalized Anxiety Disorder
HABC-M: Healthy Aging Brain Care monitor
HADS: Hospital Anxiety and Depression Scale
ICU: intensive care unit
m-CCRP: mobile critical care recovery program
MMSE: Mini-mental State Examination
PHQ-9: Patient Health Questionnaire-9
RBANS: Repeatable Battery for the Assessment of Neuropsychological Status
SF-36: medical outcomes study 36-item short form
SPPB: Short Physical Performance Battery.
